# Involvement of miRNA-34a regulated Krüppel-like factor 4 expression in hyperoxia-induced senescence in lung epithelial cells

**DOI:** 10.1186/s12931-022-02263-8

**Published:** 2022-12-10

**Authors:** Hajime Maeda, Hongwei Yao, Hayato Go, Kelsey E. Huntington, Monique E. De Paepe, Phyllis A. Dennery

**Affiliations:** 1 Department of Molecular Biology, Cellular Biology, and Biochemistry, Division of Biology and Medicine, Brown University, Providence, RI USA; 2 Department of Pediatrics, Fukushima Medical University School of Medicine, Fukushima, Japan; 3 Department of Pathology and Laboratory Medicine, Warren Alpert Medical School, Brown University, Providence, RI USA; 4 Department of Pathology, Women and Infants Hospital, Providence, RI USA; 5 Department of Pediatrics, Warren Alpert School of Medicine of Brown University, Providence, RI USA

**Keywords:** Bronchopulmonary dysplasia, microRNA-34a, Senescence, Hyperoxia, Krüppel-like factor 4

## Abstract

**Background:**

Premature infants, subjected to supplemental oxygen and mechanical ventilation, may develop bronchopulmonary dysplasia, a chronic lung disease characterized by alveolar dysplasia and impaired vascularization. We and others have shown that hyperoxia causes senescence in cultured lung epithelial cells and fibroblasts. Although miR-34a modulates senescence, it is unclear whether it contributes to hyperoxia-induced senescence. We hypothesized that hyperoxia increases miR-34a levels, leading to cellular senescence.

**Methods:**

We exposed mouse lung epithelial (MLE-12) cells and primary human small airway epithelial cells to hyperoxia (95% O_2_/5% CO_2_) or air (21% O_2_/5% CO_2_) for 24 h. Newborn mice (< 12 h old) were exposed to hyperoxia (> 95% O_2_) for 3 days and allowed to recover in room air until postnatal day 7. Lung samples from premature human infants requiring mechanical ventilation and control subjects who were not mechanically ventilated were employed.

**Results:**

Hyperoxia caused senescence as indicated by loss of nuclear lamin B1, increased p21 gene expression, and senescence-associated secretory phenotype factors. Expression of miR-34a-5p was increased in epithelial cells and newborn mice exposed to hyperoxia, and in premature infants requiring mechanical ventilation. Transfection with a miR-34a-5p inhibitor reduced hyperoxia-induced senescence in MLE-12 cells. Additionally, hyperoxia increased protein levels of the oncogene and tumor-suppressor Krüppel-like factor 4 (KLF4), which were inhibited by a miR-34a-5p inhibitor. Furthermore, KLF4 knockdown by siRNA transfection reduced hyperoxia-induced senescence.

**Conclusion:**

Hyperoxia increases miR-34a-5p, leading to senescence in lung epithelial cells. This is dictated in part by upregulation of KLF4 signaling. Therefore, inhibiting hyperoxia-induced senescence via miR-34a-5p or KLF4 suppression may provide a novel therapeutic strategy to mitigate the detrimental consequences of hyperoxia in the neonatal lung.

## Introduction

Bronchopulmonary dysplasia (BPD) is the most common chronic lung disease in premature infants. Most preterm infants can survive due to medical advances [1]. However, the prevalence of BPD is increasing, most likely due to the increased survival of newborns with extremely low gestational age. BPD increases the risk of pulmonary morbidity and adverse neurodevelopmental outcome [2–5]. Risk factors for BPD are complex, including inflammation, surfactant deficiency, ventilation, and oxygen toxicity [6]. A key pathophysiological feature of BPD is alveolar dysplasia and impaired vascularization along with inflammatory responses and fibrogenesis [7].

Genomic-wide expression profiling using DNA microarrays has recently identified novel-BPD-related genes and pathways [8, 9]. A complementary approach to profile gene expression in BPD is to analyze microRNA (miRNA, miR) expression signatures [10, 11]. These miRNAs are 21–24 nucleotide non-coding RNAs that regulate gene expression through post-transcriptional repression by binding to the 3′-untranslated region (3′-UTR) of target gene mRNA or mRNA translation at the initiation, post-initiation and elongation steps [12]. They play important roles in modulating cell differentiation, proliferation, inflammation, cell death, and senescence [13–16]. Recent studies support the hypothesis that dysregulation of multiple proteins by miRNAs contributes to the pathogenesis of BPD [17–19]. Therefore, miRNAs have been considered as promising candidates for novel targeted therapeutic approaches to BPD.

Senescence is a protective response to stress in which cells acquire a non-proliferative state and develop a pro-inflammatory senescence associated secretory phenotype (SASP) that mediates paracrine effects [20]. The senescence response causes striking changes in cellular phenotype, including a permanent arrest of cell proliferation, development of resistance to apoptosis, and an altered pattern of gene expression [21]. Senescence-inducing signals usually engage either the p53-p21 or the p16-retinoblastoma protein (pRB) pathways. Developmental senescence, which allows for tissue remodeling and organ differentiation, is regulated by p21 [22]. In contrast, stress-induced senescence occurs after damage to double-stranded DNA through reactive oxygen species. This type of senescence is usually mediated by p53 and pRB but is also stress specific [23]. The p21 protein has been shown to inhibit cell proliferation thereby inducing cell senescence to allow cells to repair damaged DNA [24, 25]. A previous study, using a mouse model, reported that a high concentration of oxygen (hyperoxia) induces lung growth and cell cycle arrest and increased p21 expression [26]. We and others have shown that hyperoxia causes senescence in cultured lung epithelial, fibroblast, and smooth muscle cells [27–29]. The mechanisms underlying hyperoxia-induced senescence are not fully understood.

We and others have shown that miR-34a was the most increased lung miRNA species using a miRNA array in a mouse model of BPD [18, 30]. Moreover, increased miR-34a expression has been reported in cells isolated from tracheal aspirate of neonates who subsequently developed BPD, in the first post-natal week [19] and that increased lung miR-34a-5p impaired endothelial angiogenesis in neonatal mice exposed to hyperoxia [19]. Most miRNAs modulate senescence by directly or indirectly regulating the p53/p21 or p16/pRB pathways [14]. Moreover, previous studies show that there may be two positive feedback loops among p53, miR-34a, and miR-34a target genes during senescence [14, 31–33]. We, therefore, hypothesized that hyperoxia causes senescence in lung epithelial cells by upregulating miR-34a.

## Materials and methods

### Cell culture

Mouse lung epithelial (MLE-12) cells and human small airway epithelial cells (SAECs) were purchased from ATCC (CRL-2110, Manassas, VA, USA) and Lonza (CC-2547, Basel, Switzerland), respectively. MLE-12 cells were maintained with 5% CO_2_, at 37℃, in DMEM/F12 media containing 2% FBS, insulin (5 µg/mL), transferrin (10 µg/mL), sodium selenite (30 nM), hydrocortisone (10 nM), β-estradiol (10 nM), HEPES (10 nM), glutamine (2 mM), and 1% P/S (100 units/mL penicillin and 100 µg/mL streptomycin). SAECs were maintained with 5% CO_2_, at 37 ℃, in SABM Basal Medium (CC-3119, Lonza) and SAGM SingleQuots supplements (CC-4124, Lonza) containing bovine pituitary extract, insulin, hydrocortisone, gentamicin/amphotericin-B, retinoic acid, fatty acid-free bovine serum albumin (BSA), transferrin, triiodothyronine, epinephrine and human epidermal growth factor.

### Hyperoxic exposure

Cells at 70–80% confluence were exposed to hyperoxia (95% O_2_ and 5% CO_2_) or air (21% O_2_ and 5% CO_2_) for 24 h. Culture media were changed every 24 h. Newborn C57BL/6J mice (< 12 h old) along with their mother were exposed to room air or hyperoxia (> 95% O_2_) for 72 h in an A-chamber (BioSpherix). The dams were switched every 24 h between room air and hyperoxia to avoid lung injury. Some pups were allowed to recover in room air until postnatal day (pnd) 7.

### Lung tissues from premature infants

Human lung samples were obtained from premature infants between 23- and 29-weeks postmenstrual age, who lived 5–15 days and required mechanical ventilation (short-term), and controls were premature infants who were not mechanically ventilated and survived less than 24 h. The clinical data of these infants are shown in Table 1. These samples were described in our previous report [34].

**Table 1:** Clinical data

	Control (n = 4)	Short-term ventilated (n = 3)
Age at birth, wk*	23.8 ± 1.0	24.3 ± 0.65
Postnatal age, d	< 1	7.3 ± 3.1
Corrected age at death, wk*	23.8 ± 1.0	25.0 ± 1.0
Sex	3 M/1F	2 M/1F
Body weight at autopsy, g	622 ± 82	796 ± 181
Clinical/autopsy diagnosis	extreme prematurity + abruption (2); PROM/abruption (1); acute chorioamnionitis + sepsis (1)	early BPD with complications of prematurity, including sepsis (1), sepsis/pneumonia (1), NEC/pneumonia (1)

BPD: bronchopulmonary dysplasia; F: female; M = male; NEC: necrotizing enterocolitis; PROM: :premature rupture of membranes

Values represent means ± SD of (n) patients

*Age and corrected age reflect postmenstrual age

### Immunofluorescence staining

Cells were fixed in 10% neutral buffered formalin (#245–684, ThermoFisher Scientific, Wilmington, DE, USA) for 15 min, followed by permeabilization with 0.2% Tween-20 (BP337, fisher scientific, Waltham, MA, USA) for 15 min at room temperature. The cells were then blocked in PBS with 0.02% Tween-20 and 1% dry milk powder (M17200, Research products international, Prospect, IL, USA) for 15 min at room temperature and subsequently incubated overnight at 4 °C with an anti-lamin B1 rabbit polyclonal antibody (ab16048, Abcam, Waltham, MA, USA, 1:200 dilution) and an anti-surfactant protein c (Spc) mouse monoclonal antibody (sc-518029, Santa Cruz, Dallas, Texas, USA, 1:50 dilution). Subsequently, the cells were incubated with secondary antibodies that include an Alexa Fluor 488 Goat anti-Rabbit IgG (A11034, Invitrogen, Waltham, MA, USA, 1:250 dilution) and an Alexa Fluor 568 Goat anti-Mouse IgG (A11004, Invitrogen, 1:250 dilution) at room temperature for 1 h. Both the primary and secondary antibodies were diluted in 0.1% BSA or 0.5% milk. After washing the cells, a coverslip was placed on the cells and they were mounted with VECTASHIELD containing DAPI (H-1200, Vector Laboratories, Burlingame, CA, USA) to detect nuclei. Images were captured using a Zeiss Axiovert 200 M Fluorescence Microscope.

For lung tissues, samples were de-paraffinized, rehydrated, and subjected to heat mediated antigen retrieval in an antigen unmasking citrate-based buffer solution (H-3300-250, Vector Labs, CA, USA). Samples were then permeabilized with 0.2% Tween-20 (BP337, fisher scientific) for 15 min at room temperature. Non-specific binding was blocked in PBS with 0.02% Tween-20 and 1% dry milk powder (M17200, Research products international, Mt Prospect, IL, USA) for 15 min at room temperature, and subsequently incubated overnight at 4 °C with an anti-KLF4 goat polyclonal antibody (AF3158, Novus Biologicals, Centennial, CO, USA, 1:200 dilution), an anti-KLF4 rabbit polyclonal antibody (ab129473, Abcam, 1:200 dilution), an anti-Spc rabbit polyclonal antibody (AB3786, Millipore, Burlington, MA, USA, 1:200 dilution), an anti-Spc mouse monoclonal antibody (sc-518029, Santa Cruz, 1:50 dilution), an anti-Hopx mouse monoclonal antibody (sc398703, Santa cruz, 1:50), an anti-von Willebrand Factor (vWF) rabbit polyclonal antibody (ab6994, abcam, 1:200), or anti-vimentin rabbit monoclonal antibody (ab92547, abcam, 1:200) as primary antibodies. Subsequently, samples were incubated with secondary antibodies that included an Alexa Fluor 488 goat anti-rabbit IgG (A11034, Invitrogen, 1:250 dilution), an Alexa Fluor 488 goat anti-mouse IgG (A11001, Invitrogen, 1:250 dilution), an Alexa Fluor 594 rabbit anti-goat IgG (A21223, Invitrogen, 1:250 dilution), or an Alexa Fluor 594 goat anti-rabbit IgG (A11072, Invitrogen, 1:250 dilution) at room temperature for 1 h. Both the primary and secondary antibodies were diluted in PBS containing 0.1% BSA or 0.5% milk. After washing samples, a coverslip was placed on the samples and they mounted with VECTASHIELD with DAPI (H-1800-10, Vector Laboratories) to detect nuclei. Images were captured using a Zeiss Axiovert 200 M Fluorescence Microscope. Cells showing co-localization of Spc with KLF4 were counted in three randomly selected high-power fields (HPF) per sample, and normalized to the number of DAPI^+^ nuclei. These experiments were carried out in a single-blinded manner.

### Measurement of miRNA and mRNA levels by qRT-PCR

Total RNA was extracted using QIAzol lysis reagent and purified using the miRNeasy Mini Kit (#217084, Qiagen, Valencia, CA, USA). RNA concentrations were measured spectrophotometrically (NanoDrop One Microvolume UV–Vis Spectrophotometer, ThermoFisher Scientific). A total of 10 ng or 400 ng RNA was used for reverse transcription using the Taqman MicroRNA Reverse Transcription Kit (4366596, ThermoFisher Scientific) and Taqman Reverse Transcription Reagents (N8080234, ThermoFisher Scientific) for miRNA and mRNA, respectively. Real-time qPCR was performed using 1.5 µL and 1 µL of cDNA in a 7300 Real-time PCR System (Applied Biosystems, Waltham, MA, USA) or QuantStudio 3 (ThermoFisher Scientific) for miRNA and mRNA, respectively. All Taqman probes were purchased from ThermoFisher Scientific. Gene expression was normalized to U6 and 18s rRNA levels. Relative RNA abundance was quantified by the comparative 2^−ΔΔCt^ method. According to the manufacturer’s recommendations, the TaqMan assay IDs were 001973 (U6 snRNA), 000426 (miR-34a-5p), Hs99999901_s1 (18 S), Mm01721290_g1 (Trp53), Mm04205640_g1 (Cdkn1a), Mm0049449_m1 (Cdkn2a), Mm00434228_m1 (Il1b), Mm00436450_m1 (Cxcl2), Mm00435858_m1 (Serpine1), and Mm00516104_m1 (Klf4) (Applied Biosystems).

### Luminex multiplex immunoassay

Cytokine levels in cell culture supernatants were measured using a custom R&D systems Murine Premixed Multi-Analyte Kit (R&D Systems, Inc., Minneapolis, MN) and a Luminex 200 Instrument (Luminex Corporation, Austin, TX) according to the manufacturer’s instructions. Samples were stored at -80 °C until the day of analysis when they were then thawed and centrifuged to pellet debris. Levels of TNF-α, IL-1α, IL-1β, Cxcl2/MIP-2, IL-10, Serpin E1/PAI-1, TNFSF12/Tweak, Cxcl12, MMP-8, MMP-12, and TNFRII/TNFRSF1B were measured. Samples were run in duplicate and analyte concentrations were reported in picograms per milliliter (pg/mL). Cytokine concentrations were calculated using a six-point standard curve derived from measurement of serially-diluted panel-specific standards run in duplicate. Panel-specific standards were used to determine analyte upper and lower limits of detection based on individual analyte standard curves.

### Detection of proteins by Western blot

Cells or lung tissues were homogenized with a RIPA buffer plus protease inhibitor cocktail (P8340, Sigma-Aldrich, St. Louis, MO, USA) and a phosphatase inhibitor cocktail set I (#524624, Sigma-Aldrich) using a tissue homogenizer for 5 s at 25,000 rpm (IKA; T25 digital Ultra Turrax, Staufen, Germany). Protein concentration was determined with the Pierce BCA assay kit (#23225, ThermoFisher Scientific). Proteins (20 μg) were separated using a NuPAGE™ 4–12% Bis-Tris protein gel (NP0335OX, Invitrogen) and transferred to PVDF membranes (IPVH00010, Millipore, Burlington, MA, USA). The membranes were blocked for 1 h at room temperature with 5% milk and then probed with primary antibodies against p53 (ab131442, Abcam), p21 (ab109199, Abcam), p16 (ab108349, Abcam), MDM4 (ab222905, Abcam or sc-74468, Santa Cruz), E2F1 (ab137415, Abcam), E2F3 (sc-56665, Santa Cruz), CCND1 (ab134175, Abcam), KLF4 (ab129473, Abcam), β-actin (ab8227, Abcam) and calnexin (ADI-SPA-860-F, Enzo, New York, NY, USA) to determine the corresponding proteins. Horseradish peroxidase conjugated secondary antibodies were used for visualization with Luminate Crescendo (WBLUR0500, EMD Millipore, St. Louis, MO, USA) or SuperSignal™ West Femto Maximum Sensitivity Substrate (#34095, ThermoFisher Scientific) using the ChemiDoc Touch Imaging System (BioRAD, Hercules, CA, USA). Equal loading of the samples was determined by quantification of proteins as well as by reprobing membranes for the reference controls β-actin or calnexin.

### Lung miR-34a detection by in situ hybridization

An miRNAScope HD (RED) Assay (ACDBio, Newark, CA, USA) was performed as previously described with modifications for miRNA [35]. In brief, lung sections were deparaffinized using xylene followed by incubation with hydrogen peroxide to block endogenous peroxidase activity. The slides were incubated with a heat-mediated target retrieval solution to unmask the target miRNA and protein. Lung sections were stained overnight at 4 ℃ with a Spc antibody (AB3786, Millipore, at a 1:200 dilution or sc-518029, Santa Cruz, 1:50 dilution). After fixing with 10% neutral buffered formalin and incubation with RNAscope protease III, a scramble negative control probe, an RNU6 positive control probe, and a mmu-miR-34a-5p probe were added to lung slides and incubated at 40 ℃ for 2 h using the HybEZ oven (ACDBio). Signals were amplified by six amplifiers with sequential hybridization of miRNAscope HD AMP 1 to AMP 6. The amplified signal was detected using an miRNAscope HD Fast Red (1:60 dilution) followed by incubation with Alexa Fluor-conjugated anti-rabbit or anti-mouse secondary antibody 488 (1:250 dilution) for 30 min at room temperature. After the staining was completed, slides were washed with PBS containing 0.1% Tween 20 and mounted with VECTASHIELD containing DAPI (H-1800-10, Vector Laboratories). Images were taken using a Zeiss Axiovert 200 M Fluorescence Microscope. Cells showing co-localization of pro-Spc with miR-34a-5p were counted and normalized to the number of DAPI^+^ nuclei. These experiments were carried out in a single-blinded manner.

### In vitro miR-34a inhibitor transfection

MLE-12 cells were transfected with a mixture of Lipofectamine RNAiMAX Transfection Reagent (#13778-150, Invitrogen), mmu-miR-34a-5p mirVana miRNA inhibitor (miR-34a inhibitor, 20 nM) (#4464084, MC11030, MIMAT0000542, ThermoFisher Scientific) or mirVana miRNA inhibitor as a negative control (negative control, 20 nM) (#4464076, ThermoFisher Scientific). The complexes were mixed in Opti-MEM™ I Reduced Serum Medium (#31985070, ThermoFisher Scientific) for 5 min prior to transfection and added directly to MLE-12 cells in culture medium grown in 6 well plates or 60 mm×15 mm dishes. After 24 h, transfected cells were used for experiments.

### In vitro KLF4 siRNA treatment

MLE-12 cells were transfected with a mixture of Lipofectamine RNAiMAX Transfection Reagent (#13778-150, Invitrogen) and Klf4 Silencer® Select (Klf4 siRNA, 30nM) (#490771, siRNA ID, s68837, ThermoFisher Scientific), or Silencer Select Negative Control No, 1 siRNA (negative control, 30 nM) (#4390843, ThermoFisher Scientific). The complexes were mixed in Opti-MEM™ I Reduced Serum Medium (#31985070, ThermoFisher Scientific) for 5 min prior to transfection and added directly to MLE-12 cells in culture medium grown in a 60 mm×15 mm dish. After 24 h, transfected cells were used for experiments.

### Statistics

Statistical analyses were performed using GraphPad Prism 8 (GraphPad Inc.). Results are expressed as mean ± SEM of individual samples. Two-group comparisons were analyzed by unpaired Student’s *t*-test, and multiple-group comparisons were performed using a one-way ANOVA followed by a Tukey’s post hoc analysis. Statistical significance was accepted at *p* < 0.05 vs. controls.

## Results

### Hyperoxia causes senescence in both MLE-12 cells and SAECs

**Fig. 1:**
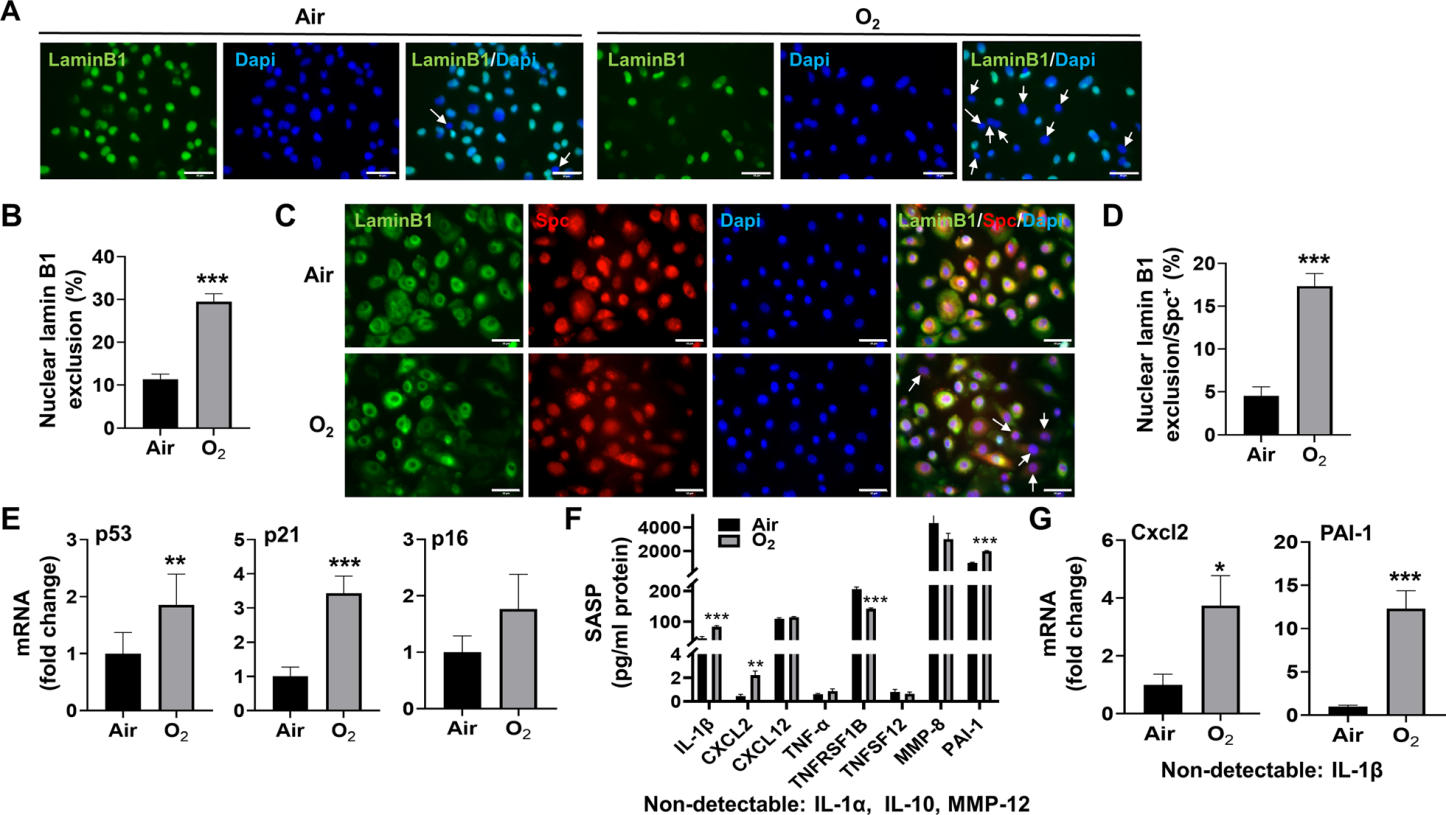
Hyperoxia causes senescence in cultured lung epithelial cells. MLE-12 cells and human SAECs were exposed to 21% O_2_/5% CO_2_ (Air) or 95% O_2_/5% CO_2_ (O_2_) for 24 h. **A**, **B** Senescence was measured by the nuclear lamin B1 loss using immunofluorescence in MLE-12 cells. The number of nuclear lamin B1 exclusion was counted in two randomly selected HPF in each sample (n = 12). **C**, **D** Senescence levels in Air and O_2_ were measured by the nuclear lamin B1 loss using immunofluorescence in SAECs. The number of nuclear lamin B1 exclusion was counted in two randomly selected HPF in each sample (n = 12). For immunofluorescence, Blue = DAPI, nuclei; Green = nuclear lamin B1; Red = Spc. The arrows indicate the nuclear lamin B1 loss. Bar size: 50 μm. **E** Transcription levels of p53, p21, and p16 were measured by RT-qPCR in MLE-12 cells (n = 9). **F** Luminex analysis was performed to evaluate soluble protein levels of SASP factors in MLE-12 cell culture supernatants (n = 5). (**G**) Transcription levels of IL-1β, Cxcl2, and PAI-1 were measured by RT-qPCR in MLE-12 cells (n = 9). Data are expressed as mean ± SEM. **p < 0.05, **p < 0.005, ***p < 0.001 versus Air group, RT-qPCR, quantitative real-time PCR

Nuclear lamin B1 exclusion can serve as a biomarker of senescence both in vitro and in vivo [36]. Using immunofluorescence of lamin B1 in MLE-12 cells and SAECs exposed to hyperoxia, we observed that 29.5% and 17.4% of cells respectively, excluded lamin B1, whereas in air exposed controls, 11.4% and 4.53% of these cells lacked lamin B1 (Fig. 1A–D). We also measured p53, p21, and p16 gene transcripts, which are senescence-associated markers. In MLE-12 cells, Taqman assay revealed significant upregulation of p53 and p21 mRNA in hyperoxia group compared to air controls, while p16 mRNA levels were unchanged between hyperoxia-exposed cells and air controls (Fig. 1E). Furthermore, we measured SASP soluble protein levels in the supernatants of the MLE12 cells. Luminex analysis revealed that the protein levels of IL-1β, Cxcl2, and PAI-1 were significantly increased, while the level of TNFRSF1B was decreased in the supernatants of the MLE-12 cells exposed to hyperoxia compared to air (Fig. 1F). In corroboration with the protein levels, the mRNA levels of the Cxcl2 and PAI-1 were significantly increased by hyperoxia in MLE-12 cells whereas IL-1β mRNA was undetectable in both hyperoxia-exposed cells and air controls (Fig. 1G). These data suggest that hyperoxia induces senescence in cultured lung epithelial cells by mechanisms not involving p16 and that hyperoxia modulates specific SASP markers.

### Hyperoxia causes senescence in newborn mouse lungs

**Fig. 2:**
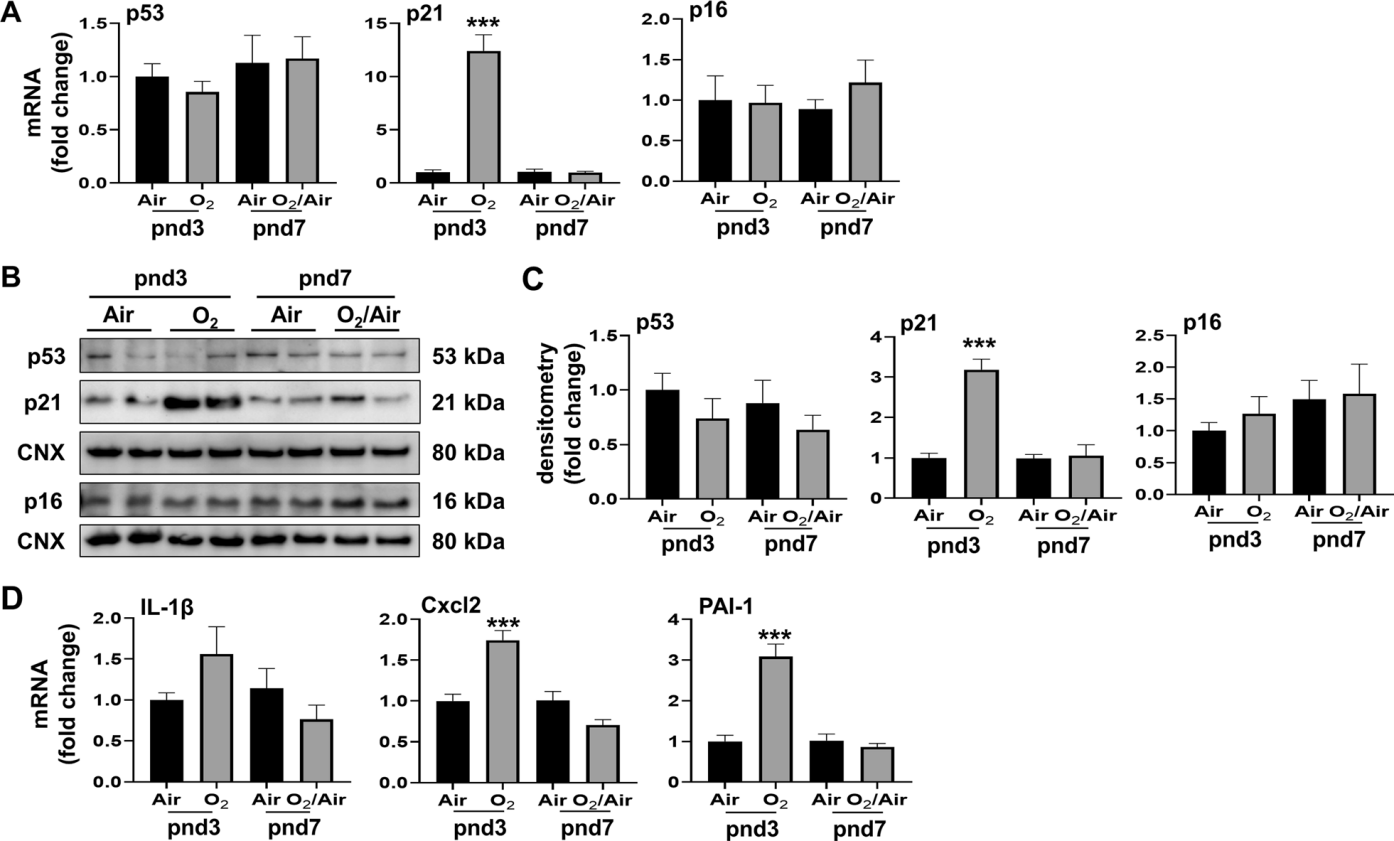
Hyperoxia causes senescence in mouse newborn lungs. Newborn C57BL/6J mice (< 12 h old) were exposed to 21% O_2_/5% CO_2_ (Air) or 95% O_2_/5% CO_2_ (O_2_) for 3 days and then allowed to recover in the air until pnd 7. **A** Transcription levels of p53, p21 and p16 were measured by RT-qPCR. **B** p53, p21, and p16 protein levels were measured by Western blot. Calnexin (CNX) was used as a loading control. **C** Densitometry analysis of p53, p21, and p16 levels after normalization. **D** Transcription levels of IL-1β, Cxcl2, and PAI-1 were measured by RT-qPCR. Data are expressed as mean ± SEM (n = 6). ***p < 0.001 versus Air group at pnd3, RT-qPCR, quantitative real-time PCR

To extrapolate above in vitro findings to an in vivo mouse model of BPD, we evaluated p21, p53 and p16 gene expression in the lung of mice exposed to hyperoxia for 3 days as neonates. As with the cultured cells, lung p21 mRNA was increased at pnd3 in mice exposed to hyperoxia as neonates, whereas p53 and p16 mRNA levels were unchanged by hyperoxia compared to the air-exposed control group (Fig. 2A). Similarly, lung p21 protein was increased at pnd3 in mice exposed to hyperoxia, whereas p53 and p16 protein levels were unchanged (Fig. 2B, C). Additionally, as with the cultured cells exposed to hyperoxia, mRNA levels for the SASP markers Cxcl2 and PAI-1 were increased in mice exposed to hyperoxia at pnd3 (Fig. 2D). However, this increase was not observed at pnd7. As with cells in culture, IL-1β mRNA levels were unchanged in lung homogenates between air and hyperoxia groups at any time point (Fig. 2D). Overall, these data indicate that hyperoxia causes senescence in newborn mouse lungs by upregulating the p21 pathway, and leads to increased expression of specific SASP markers.

### Lung miR-34a-5p is upregulated in newborn mice, MLE-12 cells, and SAECs exposed to hyperoxia, and in premature infants requiring mechanical ventilation

**Fig. 3:**
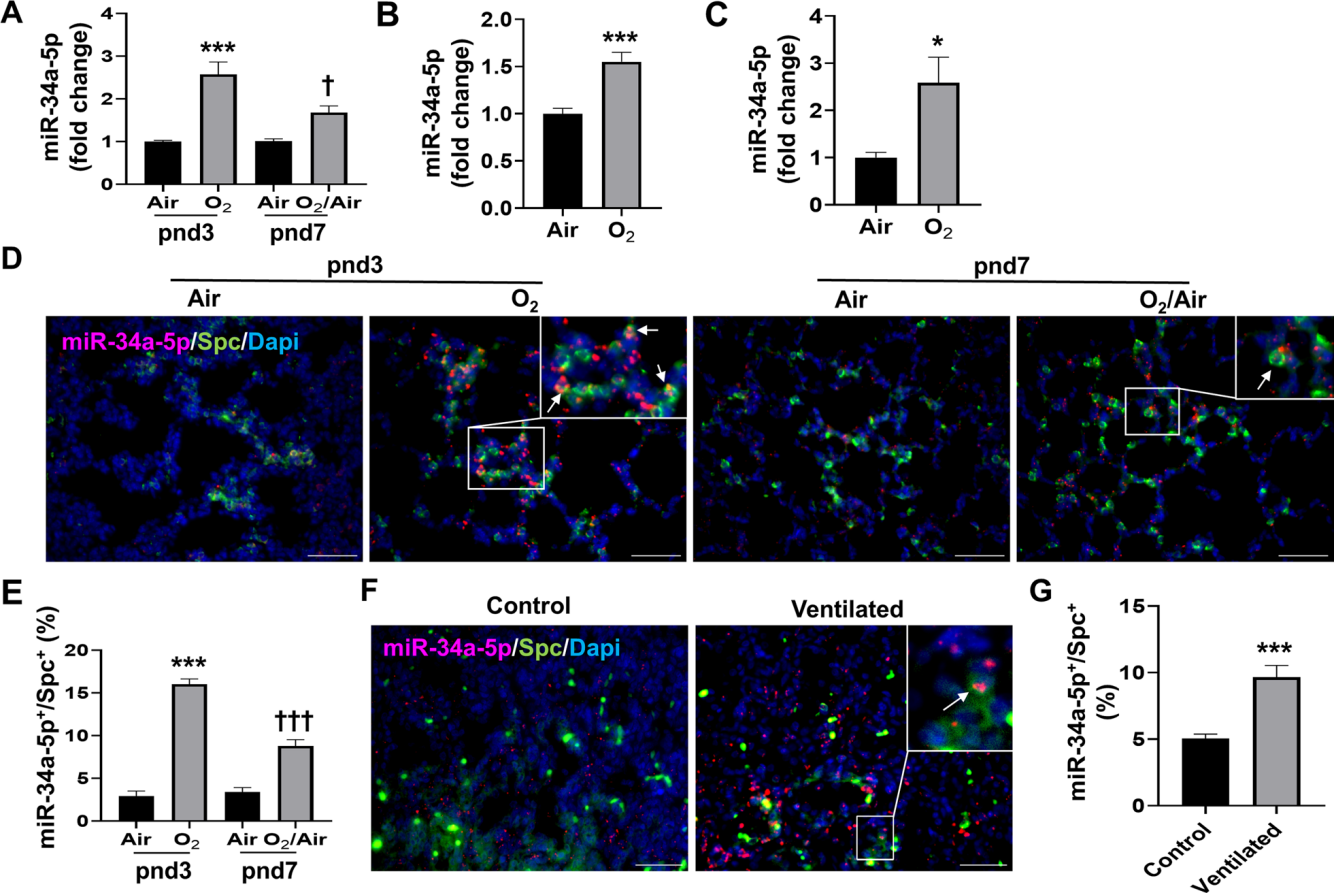
Hyperoxia up-regulates miR-34a-5p in mouse newborn lungs and in cultured lung epithelial cells. Newborn C57BL/6J mice (< 12 h old) were exposed to 21% O_2_/5% CO_2_ (Air) or 95% O_2_/5% CO_2_ (O_2_) for 3 days and then allowed to recover in the air until pnd 7. MLE-12 cells and human SAECs were exposed to Air or O_2_ for 24 h. **A** miR-34a-5p expression was measured by qPCR in mouse newborn lungs (n = 6). **B** miR-34a-5p expression was measured by qPCR in MLE-12 cells (n = 9). **C** miR-34a-5p expression was measured by RT-qPCR in SAECs (n = 8–9). **D**, **E** miRNAscope and immunofluorescence were performed to detect co-localization of miR-34a-5p with Spc. The number of co-localized cells was counted in three randomly selected HPF in each mouse (n = 3). This was normalized to Dapi + cells. Bar size: 50 μm. **F**, **G** miRNAscope and immunofluorescence were performed to detect miR-34a-5p with Spc in the lungs of premature infants requiring mechanical ventilation. The number of co-localized cells was counted in three randomly selected HPF per sample (n = 3). This was normalized to Dapi + cells. Bar size: 50 μm. Data are expressed as mean ± SEM. *p < 0.05, ***p < 0.001 versus Air group at pnd3, ^†^*p* < 0.05, ^†††^*p* < 0.001 versus Air group at pnd7, RT-qPCR, quantitative real-time PCR

Although neonatal hyperoxia increases miR-34a in the lung [19], it is unclear which lung cells are targeted. Here, we assessed whether hyperoxia upregulates miR-34a in lung epithelial cells. Hyperoxia significantly increased miR-34a-5p expression in the lung of neonatal mice (2.6-fold and 1.7-fold increase at pnd3 and pnd7, respectively, Fig. 3A), as well as in MLE-12 cells (1.6-fold, Fig. 3B) and SAECs (2.6-fold, Fig. 3C). In addition, dual immunofluorescence showed that the number of miR-34a-5p^+^/Spc^+^ cells were increased at pnd3 and pnd7 in the lungs of mice exposed to hyperoxia compared to corresponding air controls (Fig. 3D and E), despite miR-34a-5p was also expressed in other cells. Finally, the number of miR-34a-5p^+^/Spc^+^ cells was significantly increased in the lungs of premature human infants requiring mechanical ventilation compared to non-ventilated controls (Fig. 3F and G). These results suggest that miR-34a-5p expression is increased in the lung of hyperoxia-exposed mice and cultured lung epithelial cells as well as in the lung of premature infants requiring ventilation.

### miR-34a-5p inhibitor reduces hyperoxia-induced senescence in MLE-12 cells

**Fig. 4:**
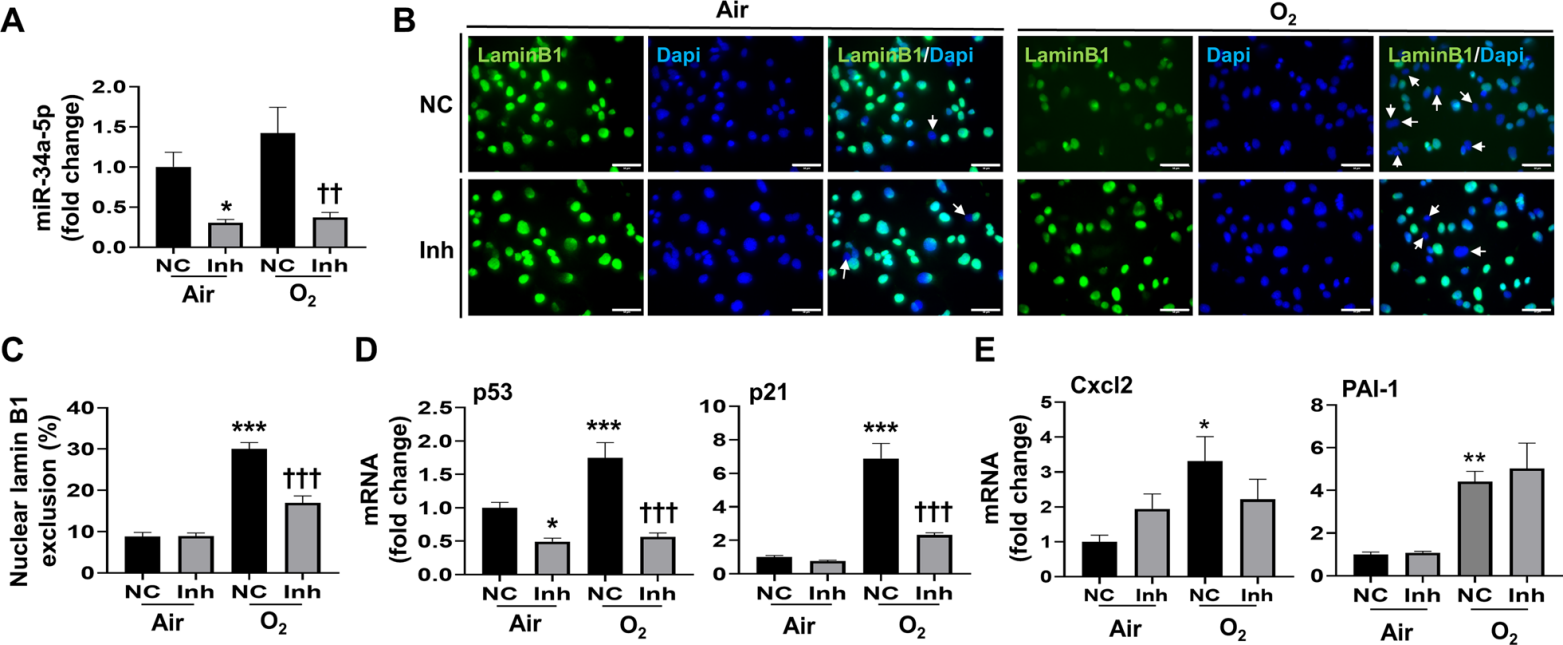
miR-34a-5p inhibitor reduces hyperoxia-induced senescence in cultured lung epithelial cells. MLE-12 cells were transfected with miR-34a-5p inhibitor (Inh) or negative control (NC) at 20 nM. 24 h after transfection, MLE-12 cells were exposed to 21% O_2_/5% CO_2_ (Air) or 95% O_2_/5% CO_2_ (O_2_) for 24 h. For immunofluorescence, Blue = DAPI, nuclei; Green = nuclear lamin B1. Bar size: 50 μm. **A** miR-34a-5p expression was measured by RT-qPCR (n = 10–11). **B**, **C** Senescence levels in Air and O_2_ were measured by the nuclear lamin B1 exclusion using immunofluorescence. The number of nuclear lamin B1 exclusion was counted in two randomly selected HPF in each sample (n = 12). **D** Transcription levels of p53 and p21 were measured by RT-qPCR (n = 10–11). **E** Transcription levels of Cxcl2 and PAI-1 were measured by RT-qPCR (n = 10–11). Data are expressed as mean ± SEM. *p < 0.05, **p < 0.01, ***p < 0.001 versus NC in Air group, ^††^*p* < 0.005, ^†††^*p* < 0.001 versus NC in O_2_ group, RT-qPCR, quantitative real-time PCR

To determine the role of miR-34a in modulating hyperoxia-induced senescence, we transfected MLE-12 cells with a miR-34a-5p inhibitor and determined senescence indicators after hyperoxic exposure. First, we verified that transfection of the miR-34a-5p inhibitor suppressed miR-34a-5p expression (Fig. 4A). Then we observed that hyperoxia-induced nuclear lamin B1 loss was reduced from 30.1 to 16.9% after transfection with the miR-34a-5p inhibitor. In contrast, under normoxic conditions, the miR-34a-5p inhibitor had no significant effects on lamin B1 expression (Fig. 4B, C). Additionally, we found that hyperoxia-induced upregulation of p53 and p21 mRNA was reduced after transfection with the miR-34a-5p inhibitor, whereas under normoxic conditions the miR-34a-5p inhibitor repressed p53 but not p21 gene expression (Fig. 4D). The hyperoxia-induced increase in Cxcl2 and PAI-1 gene expression was unchanged after transfection with the miR-34a-5p inhibitor (Fig. 4E). Overall, these results demonstrate that the miR-34a-5p inhibitor partially reduces hyperoxia-induced senescence and senescence signaling in MLE-12 cells.

### Hyperoxia increases KLF4 protein expression which is reduced by transfection with a miR-34a-5p inhibitor in MLE-12 cells

**Fig. 5:**
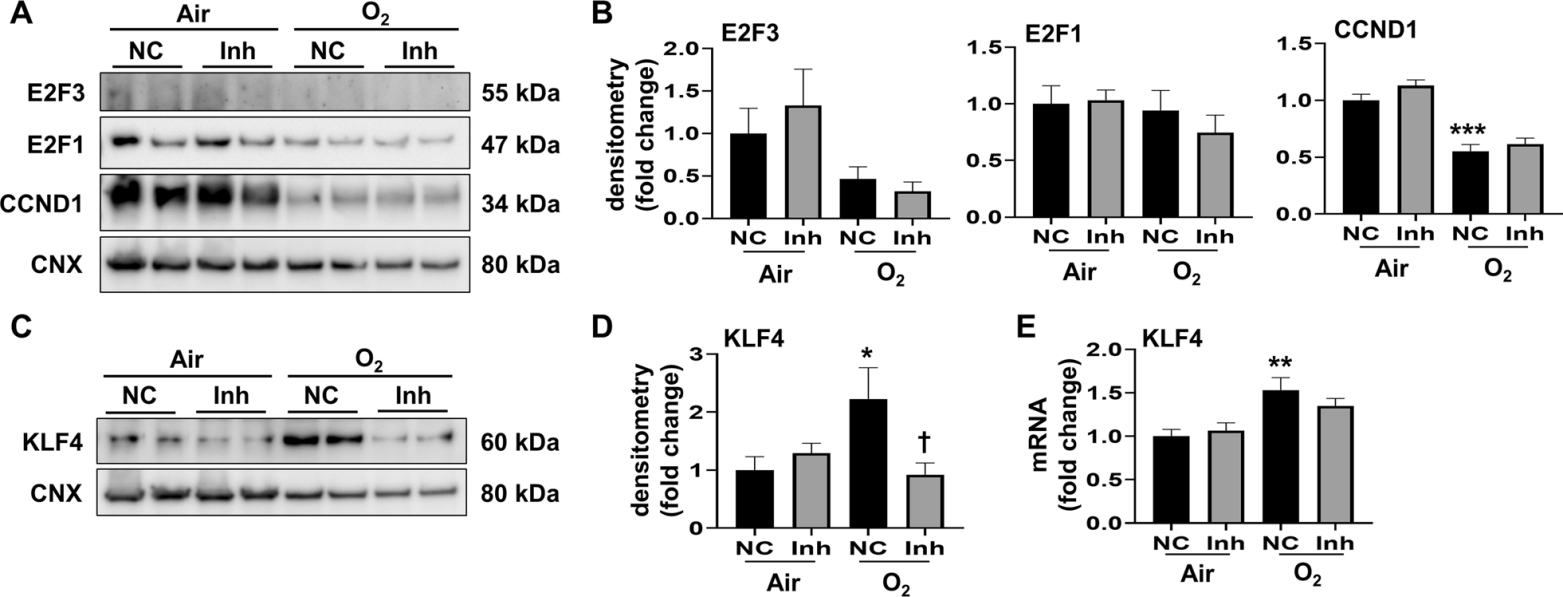
miR-34a-5p inhibitor reduces hyperoxia-induced KLF4 protein expression in cultured lung epithelial cells. MLE-12 cells were transfected with miR-34a-5p inhibitor (Inh) or negative control (NC) at 20 nM. 24 h after transfection, MLE-12 cells were exposed to 21% O_2_/5% CO_2_ (Air) or 95% O_2_/5% CO_2_ (O_2_) for 24 h. **A–D** E2F3, E2F1, CCND1, and KLF4 protein levels were measured by Western blot. Densitometry of bands was normalized using calnexin (CNX) levels. **E** Transcription levels of Klf4 were measured by RT-qPCR. Data are expressed as mean ± SEM (n = 6). ^*^p < 0.05, **p < 0.01, ***p < 0.001 versus air NC, group, †*p* < 0.05 versus NC in O_2_ group, RT-qPCR, quantitative real-time PCR

To determine the downstream signals of miR-34a-5p, we used bioinformatics tools (i.e., Targetscan and miRDB) to identify the regulators of senescence. We predicted possible miR-34a-5p downstream molecules, including MDM4, E2F3, CCND1, and KLF4, as they had a conserved miR-34a-5p seed sequence in their 3’UTR. The expression level of MDM4, E2F3, E2F1, and CCND1 were first evaluated in MLE-12 cells exposed to hyperoxia transfected with a miR-34a-5p inhibitor. We were unable to detect MDM4 protein in cells in hyperoxia with or without miR-34a inhibitor transfection (data not shown). After hyperoxia, E2F3 and E2F1 were unchanged, whereas CCND1 was decreased in MLE-12 cells. However, transfection with the miR-34a-5p inhibitor did not change the levels of these proteins in cells exposed to hyperoxia (Fig. 5A and B).

Nevertheless, in MLE-12 cells transfected with the miR-34a-5p inhibitor control, KLF4 protein levels were significantly increased by hyperoxic exposure (Fig. 5C). Conversely, the miR-34a-5p inhibitor significantly reduced hyperoxia-induced KLF4 protein levels, but not mRNA (Fig. 5C–E). These data suggest that KLF4 is a functional downstream molecule of miR-34a-5p during hyperoxia-induced senescence in lung epithelial cells, and that the mechanism by which miR-34a inhibitor suppresses KLF4 is via post-transcriptional mechanisms.

### Klf4 siRNA knockdown reduces hyperoxia-induced senescence in MLE-12 cells

**Fig. 6:**
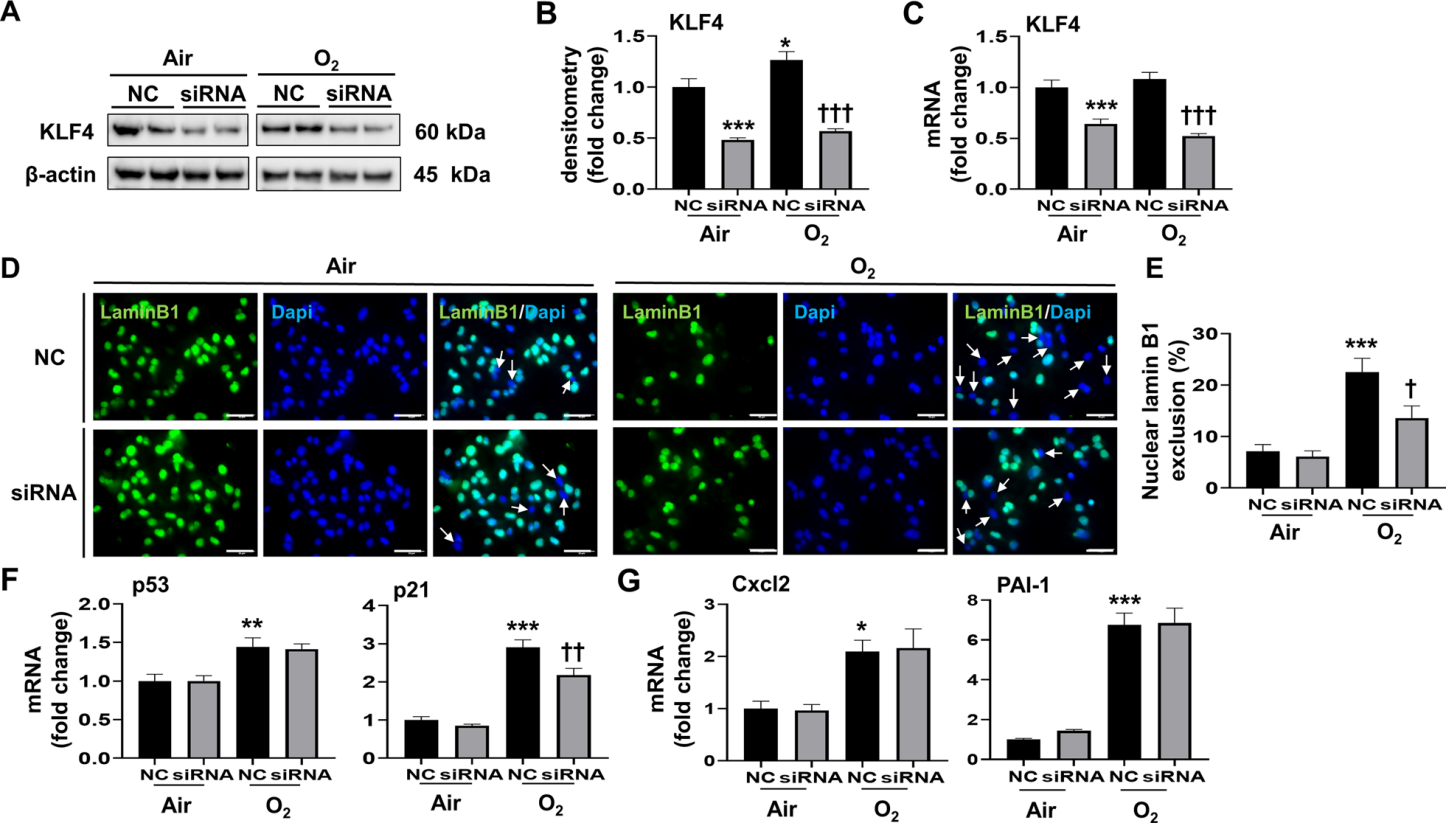
Klf4 siRNA reduces hyperoxia-induced senescence in cultured lung epithelial cells. MLE-12 cells were transfected with Klf4 siRNA or negative control (NC) at 30nM. At 24 h after transfection, MLE-12 cells were exposed to 21% O_2_/5% CO_2_ (Air) or 95% O_2_/5% CO_2_ (O_2_) for 24 h. For immunofluorescence, Blue = DAPI, nuclei; Green = nuclear lamin B1. **A**, **B** KLF4 protein levels were measured by Western blot. Densitometry of bands was normalized using β-actin levels (n = 9–10). **C** Transcription levels of Klf4 were measured by RT-qPCR (n = 9). **D**, **E** Senescence levels in Air and O_2_ were measured by the nuclear lamin B1 exclusion using immunofluorescence. The number of nuclear lamin B1 exclusion was counted in two randomly selected HPF in each sample (n = 12). **F** Transcription levels of p53 and p21 were measured by RT-qPCR (n = 9). **G** Transcription levels of Cxcl2 and PAI-1 were measured by RT-qPCR (n = 9). Data are expressed as mean ± SEM. *p < 0.05, **p < 0.01, ^***^p < 0.001 versus NC in Air group, ^†^*p* < 0.05, ^††^*p* < 0.01, ^†††^*p* < 0.001 versus NC in O_2_ group, RT-qPCR, quantitative real-time PCR

To determine the role of KLF4 in mediating hyperoxia-induced senescence, we transfected MLE-12 cells with Klf4 siRNA and evaluated senescence indicators. Transfection of the Klf4 siRNA suppressed KLF4 protein and mRNA expression (Fig. 6A–C). In addition, hyperoxia-induced nuclear lamin B1 loss was reduced from 22.5 to 13.6% after transfection with Klf4 siRNA, while Klf4 siRNA had no such effect under normoxic conditions (Fig. 6D and E). Moreover, we found that hyperoxia-induced up-regulation of p21 mRNA was reduced after transfection with Klf4 siRNA, whereas these effects were unchanged by Klf4 siRNA transfection under normoxic conditions (Fig. 6F). Nevertheless, hyperoxia-induced up-regulation of Cxcl2 and PAI-1 mRNA was unchanged after transfection with Klf4 siRNA (Fig. 6G). These results suggest that increased KLF4 contributes to hyperoxia-induced senescence in MLE-12 cells.

### KLF4 protein expression is increased in the lung of hyperoxia-exposed mice and premature infants requiring mechanical ventilation

**Fig. 7:**
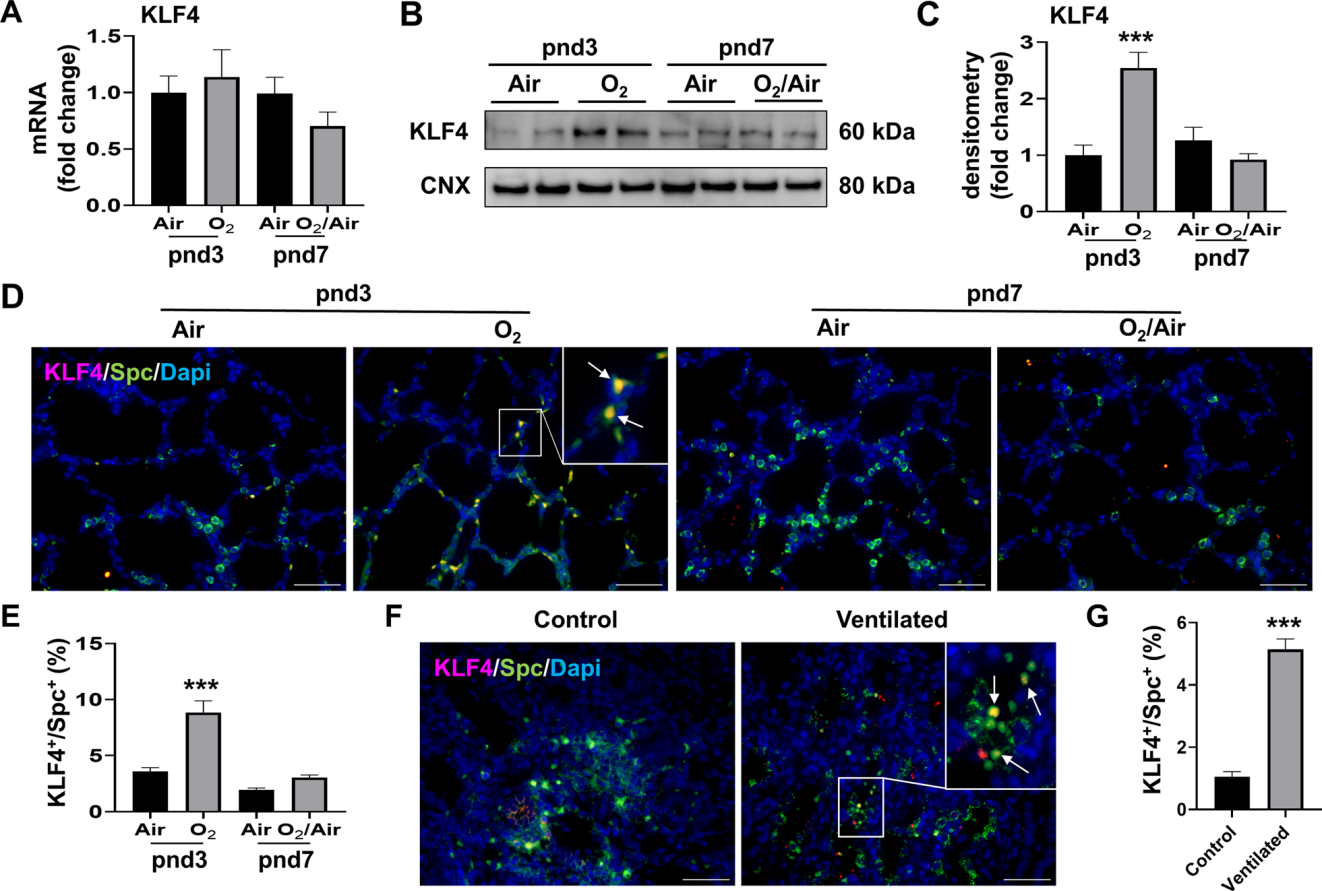
KLF4 protein expression is increased in the lung of mice exposed to hyperoxia and premature infants requiring mechanical ventilation. Newborn C57BL/6J mice (< 12 h old) were exposed to 21% O_2_/5% CO_2_ (Air) or 95% O_2_/5% CO_2_ (O_2_) for 3 days and then allowed to recover in the air until pnd 7. **A** Transcription levels of Klf4 were measured by RT-qPCR. **B**, **C** KLF4 protein levels were measured by Western blot. Densitometry of bands was normalized using calnexin (CNX) levels. **D**, **E** Double immunofluorescence was performed to detect co-localization of KLF4 with Spc. The number of co-localized cells was counted in three randomly selected HPF in each mouse (n = 3). This was normalized to Dapi^+^ cells. Bar size: 50 μm. **F**, **G** Double immunofluorescence were performed to detect co-localization of KLF4 with Spc in the lungs of premature infants requiring mechanical ventilation. The number of co-localized cells was counted in three randomly selected HPF per sample (n = 3). This was normalized to Dapi + cells. Bar size: 50 μm. Data are expressed as mean ± SEM. ***p < 0.001 versus air group at pnd3

**Fig. 8:**
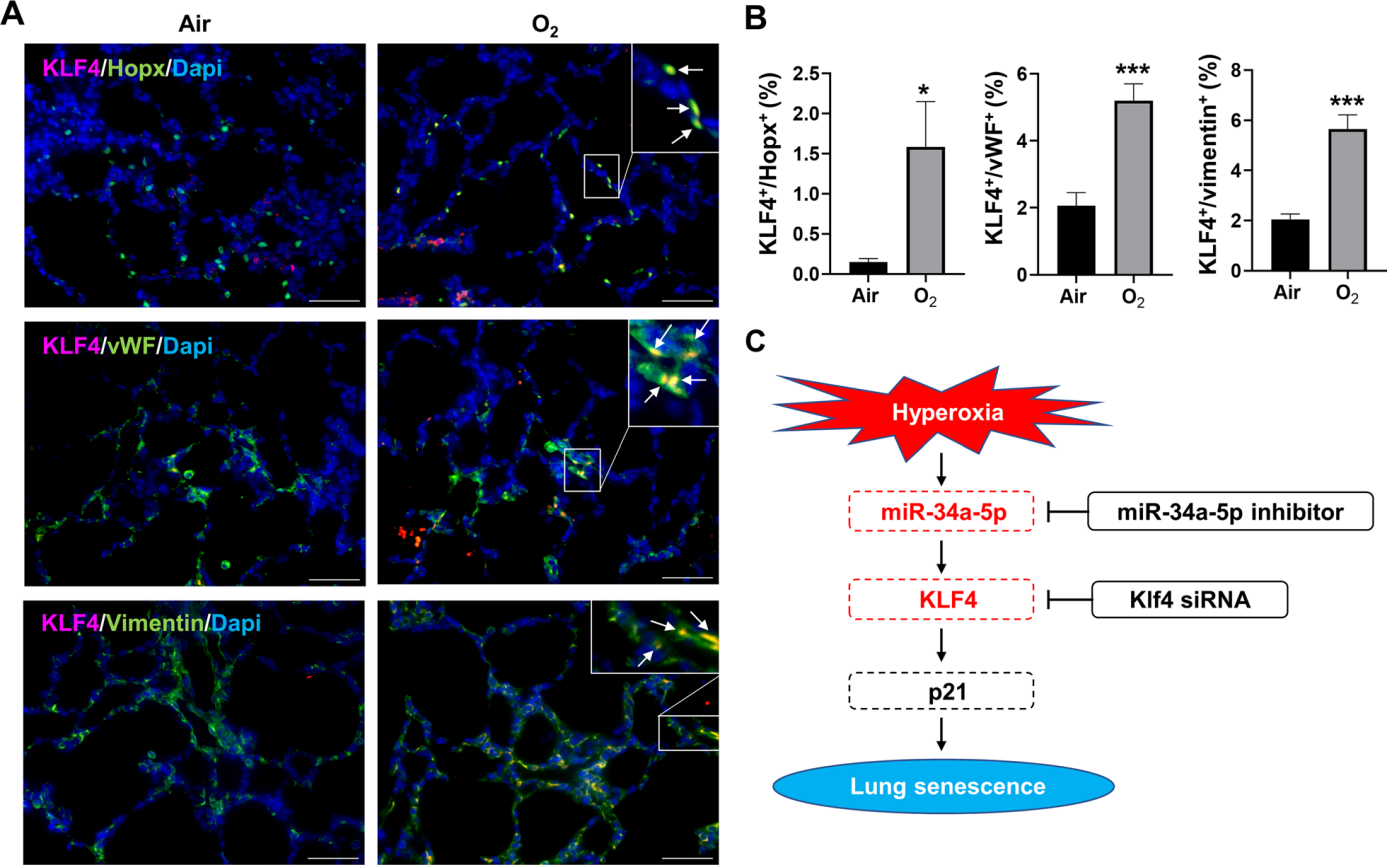
KLF4 protein expression is increased in AT1, endothelia and mesenchymal cells in the hyperoxia-exposed lung. Newborn C57BL/6J mice (< 12 h old) were exposed to 21% O_2_/5% CO_2_ (Air) or 95% O_2_/5% CO_2_ (O_2_) for 3 days. **A**, **B** Dual immunofluorescence was performed to detect co-localization of KLF4 with Hopx, Von Willebrand Factor (vWF), or vimentin. The number of co-localized cells was counted in three randomly selected HPF in each mouse (n = 3). This was normalized to Dapi^+^ cells. Bar size: 50 μm. **C** Schematic figure showing hyperoxia-induced senescence via miR-34a/KLF4/p21 pathway. Data are expressed as mean ± SEM. *p < 0.05, ***p < 0.001 versus air group

To further determine whether hyperoxia increases KLF4 expression in vivo, we measured KLF4 protein expression in lung AT2 cells after neonatal hyperoxia in newborn mice. Hyperoxia significantly increased KLF4 protein expression in the lung of neonatal mice exposed to hyperoxia at pnd3, whereas neonatal hyperoxia did not change Klf4 mRNA expression at pnd3 or pnd7 (Fig. 7A–C). Furthermore, we found that the numbers of KLF4^+^/Spc^+^, KLF4^+^/Hopx^+^, KLF4^+^/vWF^+^, and KLF4^+^/vimentin^+^ cells were increased at pnd3 in hyperoxia compared to air controls (Figs. 7D and E and 8A and B). Finally, the number of KLF4^+^/Spc^+^ cells was significantly increased in the lungs of premature human infants requiring mechanical ventilation compared to non-ventilated controls (Fig. 7F and G). These results suggest that KLF4 protein is expressed in many cell types, including AT1, AT2, endothelial and mesenchymal cells, in the hyperoxia-exposed mouse lung, and that it regulates senescence signaling.

## Discussion

The present study reports two significant and novel findings. First, hyperoxia increases the expression of miR-34a-5p in cultured lung epithelial cells and in mouse lung tissues and that miR-34a-5p regulates hyperoxia-induced senescence in lung epithelial cells. Secondly, KLF4 is a downstream signal of miR-34a-5p, which mediates hyperoxia-induced senescence in lung epithelial cells. These results indicate the potential therapeutic value of miR-34a-5p inhibitors in the treatment of BPD.

Several studies have shown that hyperoxia causes senescence in cultured lung epithelial, fibroblasts, and smooth muscle cells [27–29]. However, there are no unique biomarkers of senescence. Senescent cells are generally identified by a combination of indicators including an absence of proliferation markers, loss of the nuclear lamina protein lamin B1, increased senescence-associated β galactosidase, increased expression of cell-cycle inhibitors, such as the cyclin-dependent kinase inhibitors p21 and p16, as well as evidence of DNA damage [37]. We previously reported that hyperoxia causes senescence in MLE-12 cells [28]. This is associated with increased double-stranded DNA damage, p53 phosphorylation and nuclear localization. Furthermore, hyperoxia-induced senescence was p53-dependent, but not pRB-dependent, in these cells [28]. Nuclear lamin B1 exclusion is a robust hallmark of senescence in culture and in vivo. It serves as an easily detectable biomarker of the senescent state [36]. Here we demonstrated that hyperoxia increased nuclear lamin B1 exclusion, the expression of p53 and p21 mRNA, and specific SASP factors. These findings indicate that hyperoxia induces senescence in vitro and in vivo. We observed that p53 mRNA and protein levels were unchanged in the lung of mice exposed to hyperoxia, suggesting that hyperoxia causes a cell-specific increase of p53 expression. Interestingly, p16 mRNA was also not altered by hyperoxia in both cultured lung epithelial cells and mouse lungs, suggesting that p16 may not participate in hyperoxia-induced senescence.

BPD is characterized by a simplified alveolar epithelium, increased inflammation, dysregulated vascularization and disrupted angiogenesis as well as decreased cell proliferation, increased cell death, and possible cellular senescence with associated mitochondrial dysfunction [38]. Several studies have investigated the role of specific miRNAs in the mechanisms underlying the pathogenesis of BPD. For example, miR-34a targets the key contributing factors leading to BPD [38, 39]. Syed et al. reported that miR-34a modulates inflammatory responses, angiogenesis, proliferation, and apoptosis-related proteins, including Sirt1, Ang1, Akt, Dll1, Notch1/Notch2, and Bcl2 in AT2 cells in a mouse model of BPD [19]. Ruiz-Camp et al. reported that miR-34a targeted Pdgfra to regulate alveolar cell proliferation and alveolarization in BPD lungs [30]. Others suggested that cellular senescence is a status of irreversible growth arrest which may participate in the development of BPD [40]. The role of miR-34a in senescence has been demonstrated by several groups [41–43]. Nevertheless, it is unclear whether increased miR-34a contributes to hyperoxia-induced senescence. In the present study, we showed that hyperoxia increased senescence by upregulating miR-34a-5 in AT2 cells. Interestingly, transfection with an miR-34a inhibitor did not attenuate hyperoxia-induced Cxcl2 or PAI-1 SASP factor gene expression. This may mean that the changes in Cxcl2 and PAI-1 are not regulated by miR-34a. However, Barazzone et al. reported that lung of mice exposed to hyperoxia overproduce PAI-1 and that PAI-1 upregulation impairs fibrinolytic activity in the alveolar compartment [44]. Li et al. reported that lung of mice exposed to hyperoxia exhibits increased Cxcl2 production and airway epithelial cell apoptosis [45]. These observations are in agreement with our findings showing increased PAI-1 and Cxcl2 in the supernatants of hyperoxia-exposed MLE-12 cells. Further study is warranted to determine whether and which SASP factors secreted from senescent cells contribute to hyperoxia-induced lung injury.

To understand the downstream mechanism dictating miR-34a-5p-mediated senescence, we used the miRNA databases TargetScan and miRDB, and identified KLF4 as a senescence-associated molecule. Others have shown that KLF4 is an important target gene for miR-34a [46, 47]. It functions as an oncogene and tumor-suppressor gene, which activates the transcription of p21, a key signal in hyperoxia-mediated senescence. The latter is an important inhibitor of cell-cycle progression and a key downstream factor of multiple tumor-suppressor pathways. Ectopic expression of KLF4 results in cell-cycle arrest and its transcriptional targets are involved in cell differentiation and cell-cycle inhibition [48]. Here, the expression of KLF4 protein and mRNA were increased after hyperoxia. Transfection of miR-34a-5p inhibitor attenuated hyperoxia-induced KLF4 protein levels. We observed that KLF4 mRNA expression was unchanged by an miR-34a inhibitor after hyperoxia. Although miRNAs usually recognize complementary sequences in the 3′UTR of their target mRNAs, Vasudevan et al. reported that they also upregulate mRNA translation under growth arrest conditions [49]. Further study is warranted to determine whether miR-34a inhibitor suppresses KLF4 protein levels by downregulating its mRNA translation, or promoting its protein degradation by intermediary factors. Overall, increased miR-34a contributes to hyperoxia-induced senescence by upregulating KLF4 protein expression.

## Conclusion

Hyperoxia increased the expression of miR-34a-5p, leading to senescence by upregulating KLF4 and p21 signaling pathways in lung epithelial cells (Fig. 8C). As miR-34a-5p has been shown to have a significant role in the pathogenesis of BPD, inhibiting miR-34a-5p or associated senescence molecules such as KLF4 may be a promising therapeutic intervention for BPD.

## Data Availability

The datasets used and/or analyzed during the current study are available from the corresponding author upon reasonable request.

## Abbreviations

3′-UTR: 3′-Untranslated regions
AT2: Type II alveolar epithelial
BPD: Bronchopulmonary dysplasia
BSA: Bovine serum albumin
HPF: High-power fields
KLF4: Krüppel-like factor 4
miRNA, miR: microRNA
MLE-12: Mouse lung epithelial
pnd: Postnatal day
pRB: Retinoblastoma protein
SAECs: Human small airway epithelial cells
SASP: Senescence associated secretory phenotype
Spc: Surfactant protein c

## References

[1] Thebaud B, Goss KN, Laughon M, Whitsett JA, Abman SH, Steinhorn RH, Aschner JL, Davis PG, McGrath-Morrow SA, Soll RF, Jobe AH (2019). Bronchopulmonary dysplasia. Nat Rev Dis Primers.

[2] Fawke J, Lum S, Kirkby J, Hennessy E, Marlow N, Rowell V, Thomas S, Stocks J (2010). Lung function and respiratory symptoms at 11 years in children born extremely preterm: the EPICure study. Am J Respir Crit Care Med.

[3] Katz TA, Vliegenthart RJS, Aarnoudse-Moens CSH, Leemhuis AG, Beuger S, Blok GJ, van Brakel MJM, van den Heuvel MEN, van Kempen A, Lutterman C (2022). Severity of bronchopulmonary dysplasia and neurodevelopmental outcome at 2 and 5 years corrected age. J Pediatr.

[4] Potharst ES, van Wassenaer-Leemhuis AG, Houtzager BA, Livesey D, Kok JH, Last BF, Oosterlaan J (2013). Perinatal risk factors for neurocognitive impairments in preschool children born very preterm. Dev Med Child Neurol.

[5] Vrijlandt EJ, Boezen HM, Gerritsen J, Stremmelaar EF, Duiverman EJ (2007). Respiratory health in prematurely born preschool children with and without bronchopulmonary dysplasia. J Pediatr.

[6] Laughon M, Allred EN, Bose C, O’Shea TM, Van Marter LJ, Ehrenkranz RA, Leviton A (2009). Patterns of respiratory disease during the first 2 postnatal weeks in extremely premature infants. Pediatrics.

[7] Baraldi E, Filippone M (2007). Chronic lung disease after premature birth. N Engl J Med.

[8] Bhattacharya S, Go D, Krenitsky DL, Huyck HL, Solleti SK, Lunger VA, Metlay L, Srisuma S, Wert SE, Mariani TJ, Pryhuber GS (2012). Genome-wide transcriptional profiling reveals connective tissue mast cell accumulation in bronchopulmonary dysplasia. Am J Respir Crit Care Med.

[9] Bhattacharya S, Zhou Z, Yee M, Chu CY, Lopez AM, Lunger VA, Solleti SK, Resseguie E, Buczynski B, Mariani TJ, O’Reilly MA (2014). The genome-wide transcriptional response to neonatal hyperoxia identifies Ahr as a key regulator. Am J Physiol Lung Cell Mol Physiol.

[10] Siddaiah R, Oji-Mmuo CN, Montes DT, Fuentes N, Spear D, Donnelly A, Silveyra P (2021). MicroRNA signatures associated with bronchopulmonary dysplasia severity in tracheal aspirates of preterm infants. Biomedicines.

[11] Xing Y, Fu J, Yang H, Yao L, Qiao L, Du Y, Xue X (2015). MicroRNA expression profiles and target prediction in neonatal Wistar rat lungs during the development of bronchopulmonary dysplasia. Int J Mol Med.

[12] He L, Hannon GJ (2004). MicroRNAs: small RNAs with a big role in gene regulation. Nat Rev Genet.

[13] Ambros V (2003). MicroRNA pathways in flies and worms: growth, death, fat, stress, and timing. Cell.

[14] Liu FJ, Wen T, Liu L (2012). MicroRNAs as a novel cellular senescence regulator. Ageing Res Rev.

[15] Marques-Rocha JL, Samblas M, Milagro FI, Bressan J, Martínez JA, Marti A (2015). Noncoding RNAs, cytokines, and inflammation-related diseases. Faseb j.

[16] Schmittgen TD (2008). Regulation of microRNA processing in development, differentiation and cancer. J Cell Mol Med.

[17] Go H, La P, Namba F, Ito M, Yang G, Brydun A, Igarashi K, Dennery PA (2016). MiR-196a regulates heme oxygenase-1 by silencing Bach1 in the neonatal mouse lung. Am J Physiol Lung Cell Mol Physiol.

[18] Go H, Maeda H, Miyazaki K, Maeda R, Kume Y, Namba F, Momoi N, Hashimoto K, Otsuru S, Kawasaki Y (2020). Extracellular vesicle miRNA-21 is a potential biomarker for predicting chronic lung disease in premature infants. Am J Physiol Lung Cell Mol Physiol.

[19] Syed M, Das P, Pawar A, Aghai ZH, Kaskinen A, Zhuang ZW, Ambalavanan N, Pryhuber G, Andersson S, Bhandari V (2017). Hyperoxia causes miR-34a-mediated injury via angiopoietin-1 in neonatal lungs. Nat Commun.

[20] Coppé JP, Desprez PY, Krtolica A, Campisi J (2010). The senescence-associated secretory phenotype: the dark side of tumor suppression. Annu Rev Pathol.

[21] Campisi J, di d’Adda Fagagna F (2007). Cellular senescence: when bad things happen to good cells. Nat Rev Mol Cell Biol.

[22] Storer M, Mas A, Robert-Moreno A, Pecoraro M, Ortells MC, Di Giacomo V, Yosef R, Pilpel N, Krizhanovsky V, Sharpe J, Keyes WM (2013). Senescence is a developmental mechanism that contributes to embryonic growth and patterning. Cell.

[23] Demaria M, O’Leary MN, Chang J, Shao L, Liu S, Alimirah F, Koenig K, Le C, Mitin N, Deal AM (2017). Cellular senescence promotes adverse effects of chemotherapy and cancer relapse. Cancer Discov.

[24] McGrath-Morrow SA, Cho C, Soutiere S, Mitzner W, Tuder R (2004). The effect of neonatal hyperoxia on the lung of p21Waf1/Cip1/Sdi1-deficient mice. Am J Respir Cell Mol Biol.

[25] Yao H, Yang SR, Edirisinghe I, Rajendrasozhan S, Caito S, Adenuga D, O’Reilly MA, Rahman I (2008). Disruption of p21 attenuates lung inflammation induced by cigarette smoke, LPS, and fMLP in mice. Am J Respir Cell Mol Biol.

[26] Londhe VA, Sundar IK, Lopez B, Maisonet TM, Yu Y, Aghai ZH, Rahman I (2011). Hyperoxia impairs alveolar formation and induces senescence through decreased histone deacetylase activity and up-regulation of p21 in neonatal mouse lung. Pediatr Res.

[27] Parikh P, Britt RD, Manlove LJ, Wicher SA, Roesler A, Ravix J, Teske J, Thompson MA, Sieck GC, Kirkland JL (2019). Hyperoxia-induced Cellular Senescence in fetal airway smooth muscle cells. Am J Respir Cell Mol Biol.

[28] Scaffa AM, Peterson AL, Carr JF, Garcia D, Yao H, Dennery PA (2021). Hyperoxia causes senescence and increases glycolysis in cultured lung epithelial cells. Physiol Rep.

[29] You K, Parikh P, Khandalavala K, Wicher SA, Manlove L, Yang B, Roesler A, Roos BB, Teske JJ, Britt RD (2019). Moderate hyperoxia induces senescence in developing human lung fibroblasts. Am J Physiol Lung Cell Mol Physiol.

[30] Ruiz-Camp J, Quantius J, Lignelli E, Arndt PF, Palumbo F, Nardiello C, Surate Solaligue DE, Sakkas E, Mižíková I, Rodríguez-Castillo JA (2019). Targeting miR-34a/Pdgfra interactions partially corrects alveologenesis in experimental bronchopulmonary dysplasia. EMBO Mol Med.

[31] Welch C, Chen Y, Stallings RL (2007). MicroRNA-34a functions as a potential tumor suppressor by inducing apoptosis in neuroblastoma cells. Oncogene.

[32] Yamakuchi M, Ferlito M, Lowenstein CJ (2008). miR-34a repression of SIRT1 regulates apoptosis. Proc Natl Acad Sci U S A.

[33] Yamakuchi M, Lowenstein CJ (2009). MiR-34, SIRT1 and p53: the feedback loop. Cell Cycle.

[34] De Paepe ME, Mao Q, Powell J, Rubin SE, DeKoninck P, Appel N, Dixon M, Gundogan F (2006). Growth of pulmonary microvasculature in ventilated preterm infants. Am J Respir Crit Care Med.

[35] Scaffa A, Yao H, Oulhen N, Wallace J, Peterson AL, Rizal S, Ragavendran A, Wessel G, De Paepe ME, Dennery PA (2021). Single-cell transcriptomics reveals lasting changes in the lung cellular landscape into adulthood after neonatal hyperoxic exposure. Redox Biol.

[36] Freund A, Laberge RM, Demaria M, Campisi J (2012). Lamin B1 loss is a senescence-associated biomarker. Mol Biol Cell.

[37] Campisi J (2013). Aging, cellular senescence, and cancer. Annu Rev Physiol.

[38] Das P, Shah D, Bhandari V (2021). miR34a: a novel small molecule regulator with a big role in bronchopulmonary dysplasia. Am J Physiol Lung Cell Mol Physiol.

[39] Das P, Syed MA, Shah D, Bhandari V (2018). miR34a: a master regulator in the pathogenesis of bronchopulmonary dysplasia. Cell Stress.

[40] Zhao H, Dennery PA, Yao H (2018). Metabolic reprogramming in the pathogenesis of chronic lung diseases, including BPD, COPD, and pulmonary fibrosis. Am J Physiol Lung Cell Mol Physiol.

[41] Ito T, Yagi S, Yamakuchi M (2010). MicroRNA-34a regulation of endothelial senescence. Biochem Biophys Res Commun.

[42] Tazawa H, Tsuchiya N, Izumiya M, Nakagama H (2007). Tumor-suppressive miR-34a induces senescence-like growth arrest through modulation of the E2F pathway in human colon cancer cells. Proc Natl Acad Sci U S A.

[43] Zhao T, Li J, Chen AF (2010). MicroRNA-34a induces endothelial progenitor cell senescence and impedes its angiogenesis via suppressing silent information regulator 1. Am J Physiol Endocrinol Metab.

[44] Barazzone C, Belin D, Piguet PF, Vassalli JD, Sappino AP (1996). Plasminogen activator inhibitor-1 in acute hyperoxic mouse lung injury. J Clin Invest.

[45] Li LF, Liao SK, Ko YS, Lee CH, Quinn DA (2007). Hyperoxia increases ventilator-induced lung injury via mitogen-activated protein kinases: a prospective, controlled animal experiment. Crit Care.

[46] Chen Q, Li L, Tu Y, Zheng LL, Liu W, Zuo XY, He YM, Zhang SY, Zhu W, Cao JP (2014). MiR-34a regulates apoptosis in liver cells by targeting the KLF4 gene. Cell Mol Biol Lett.

[47] Pan Y, Hui X, Hoo RLC, Ye D, Chan CYC, Feng T, Wang Y, Lam KSL, Xu A (2019). Adipocyte-secreted exosomal microRNA-34a inhibits M2 macrophage polarization to promote obesity-induced adipose inflammation. J Clin Invest.

[48] Rowland BD, Peeper DS (2006). KLF4, p21 and context-dependent opposing forces in cancer. Nat Rev Cancer.

[49] Vasudevan S, Tong Y, Steitz JA (2007). Switching from repression to activation: microRNAs can up-regulate translation. Science.

